# Reaktionsmuster der lokoregionären Lymphknoten im Abflussgebiet von COVID-19-Lungen

**DOI:** 10.1007/s00292-021-00914-z

**Published:** 2021-02-11

**Authors:** Jasmin D. Haslbauer, Matthias S. Matter, Anna K. Stalder, Alexandar Tzankov

**Affiliations:** 1 Pathologie, Institut für Medizinische Genetik und Pathologie, Universitätsspital Basel, Universität Basel, Basel, Schweiz; 2 Institut für Medizinische Genetik und Pathologie, Schönbeinstrasse 40, 4031 Basel, Schweiz

**Keywords:** CD8-positive T‑Lymphozyten, Zytokin-Freisetzungssyndrom, Keimzentrum, Immunohistochemie, SARS-CoV‑2, CD8-positive T‑lymphocytes, Cytokine release syndrome, Germinal center, Immunohistochemistry, SARS-CoV‑2

## Abstract

**Hintergrund:**

Eine dysregulierte Immunantwort, z. B. in der Form eines Zytokinsturmes, einer Störung des Immunglobulinklassenwechsels, eines sog. antikörpervermitteltem Enhancements oder einer aberranten Antigenpräsentation wurde bereits in schweren Krankheitsverläufen von COVID-19 beschrieben.

**Ziel der Arbeit:**

Zur Charakterisierung der COVID-19-Immunantwort wurde die Histomorphologie der Lymphknoten des pulmonalen Abflussgebietes untersucht.

**Material und Methoden:**

Regionale Lymphknoten des pulmonalen Abflussgebiets wurden bei COVID-19-Autopsien asserviert (*n* = 20). Deren Histomorphologie, SARS-CoV-2-qRT-PCR sowie Genexpressionsanalysen von gängigen Genen der Immunantwort wurden berücksichtigt.

**Ergebnisse:**

Histologisch zeigten sich ein mäßig- bis schwergradiges Ödem mit Kapillarostase, eine erhöhte Anzahl von extrafollikulären Plasmablasten, milde bis mäßige Plasmazytose, vermehrte CD8^+^-T-Zellen und CD11c/CD68^+^-Histiozyten mit Hämophagozytoseaktivität. Von 20 Fällen wiesen 18 hypoplastische oder fehlende Keimzentren sowie eine Verminderung der follikulären dendritischen Zellen und follikulären T‑Helferzellen auf. In 14 von 20 Fällen war der qRT-PCR-Nachweis von SARS-CoV‑2 positiv, jedoch zeigte sich nur bei einem einzigen Fall eine immunhistochemische Positivität für SARS-CoV-2-*N*-Antigene in Sinushistiozyten. In Genexpressionsanalysen war eine erhöhte Expression von *STAT1, CD163, Granzym B, CD8A, MZB1* und *PAK1*, neben *CXCL9* zu beobachten.

**Diskussion:**

Die Befunde in den Lymphknoten deuten auf eine dysregulierte Immunantwort bei schweren COVID-19-Krankheitsverläufen hin. Insbesondere impliziert das Ausbleiben der Keimzentrumsreaktion und die vermehrte Präsenz von Plasmablasten eine nur transiente B‑Zellreaktion, welche die Entwicklung einer Langzeitimmunität infrage stellt.

## Abstract (en)

**Background:**

A dysregulated immune response is considered one of the major factors leading to severe COVID-19. Previously described mechanisms include the development of a cytokine storm, missing immunoglobulin class switch, antibody-mediated enhancement, and aberrant antigen presentation.

**Objectives:**

To understand the heterogeneity of immune response in COVID-19, a thorough investigation of histomorphological patterns in regional lymph nodes was performed.

**Materials and methods:**

Lymph nodes from the cervical, mediastinal, and hilar regions were extracted from autopsies of patients with lethal COVID-19 (*n* = 20). Histomorphological characteristics, SARS-CoV‑2 qRT-PCR, and gene expression profiling on common genes involved in immunologic response were analyzed.

**Results:**

Lymph nodes displayed moderate to severe capillary stasis and edema, an increased presence of extrafollicular plasmablasts, mild to moderate plasmacytosis, a dominant population of CD8^+^ T‑cells, and CD11c/CD68^+^ histiocytosis with hemophagocytic activity. Out of 20 cases, 18 presented with hypoplastic or missing germinal centers with a decrease of follicular dendritic cells and follicular T‑helper cells. A positive viral load was detected by qRT-PCR in 14 of 20 cases, yet immunohistochemistry for SARS-CoV-2 *N*-antigen revealed positivity in sinus histiocytes of only one case. Gene expression analysis revealed an increased expression of *STAT1, CD163, granzyme B, CD8A, MZB1,* and *PAK1,* as well as *CXCL9*.

**Conclusions:**

Taken together, our findings imply a dysregulated immune response in lethal COVID-19. The absence/hypoplasia of germinal centers and increased presence of plasmablasts implies a transient B‑cell response, implying an impaired development of long-term immunity against SARS-CoV‑2 in such occasions.

Die Coronavirus-Krankheit-2019(COVID-19)-Pandemie, verursacht durch das „severe acute respiratory syndrom coronavirus 2“ (SARS-CoV-2), dominierte das Jahr 2020 sowie die ersten Monate des laufenden Jahres. Obwohl der Ursprung des Virus, dessen zellulären Eintrittsmechanismen und die Epidemiologie rasch geklärt werden konnten [2, 18, 41], sind viele grundlegende immunpathologische Fragestellungen unbeantwortet.

So blieben *In-situ*-Analysen der viralen Interaktion mit dem menschlichen Körper für eine lange Zeit auf der Ebene von Fallberichten und kleinen Serien [43], obwohl sie – wie sich im Nachhinein herausstellte [1, 30] – entscheidende Beiträge zum Verständnis dieser neuartigen Erkrankung liefern konnten. So wurde bereits bei den ersten schwereren klinischen COVID-19-Fällen eine Lymphopenie beobachtet [16], deren Ausmaß mit dem Schweregrad der Erkrankung zusammenzuhängen schien, begleitet von einer vermehrten Anfälligkeit von bakteriellen Superinfektionen [30]. Das dazugehörige morphologische Korrelat in lymphatischen Geweben ist jedoch nicht restlos geklärt.

Die spezifischen Reaktionsmuster der Lymphknoten [42] können einen wertvollen Einblick in die immunpathologischen Mechanismen vieler Krankheitsbilder liefern. Sie sind Ausdruck komplexer Interaktionen zwischen den auslösenden Agenzien (Antigene), der Immunzellen des Wirtes, der löslichen Botenstoffe (zum Beispiel Zytokine, Chemokine und Wachstumsfaktoren) und weiterer Moleküle der Körperabwehr wie Komplementbestandteile [39]. Die Immunogenetik des Wirtes wie Genpolymorphismen der Botenstoffe, ihrer Rezeptoren und der Histokompatibilitätsgene bestimmen oft den Schweregrad und die Dauer der entsprechenden Veränderungen, wie beispielsweise bei der Sarkoidose [14]. Als Pendant einer adäquaten Immunantwort gilt die am häufigsten zu beobachtende follikuläre Hyperplasie mit damit assoziierter Produktion von höchstaffinen Antikörpern und Aufbau eines immunologischen Gedächtnisses [39]. Einige sonstige augenfällige morphologische Muster sind Ausdruck der prädominant vorherrschenden Achse der Botenstoffe und Zellen. Das Muster einer angiofollikulären (Castleman-ähnlichen) Hyperplasie ist assoziiert mit chronischer Einwirkung von Interleukin (IL) 6 [12], während Granulome mit einer Überproduktion von IL‑2, IL-12 und Tumornekrosefaktor‑α (TNF-α) [17] einhergehen. Der systemische Lupus erythematodes, die Kikuchi-Fujimoto-Erkrankung und die Toxoplasmose zeigen eine Zunahme CD123/CD303^+^ plasmazytoider dendritischer Zellen in den Lymphknoten aufgrund von Aktivierung der Toll-like-Rezeptoren 7 und 9 (TLR7, TLR9) und Überproduktion von Interferon‑γ (IFN-γ) [20].

Ein etwas weniger beachtetes Lymphknotenreaktionsmuster ist die sog. extrafollikuläre Proliferation von B‑Zellen (B-Blasten) in Abwesenheit von follikulärer Hyperplasie [5]. Es wird angenommen, dass dies das morphologische Korrelat schneller bzw. primärer und transienter B‑Zell-Expansion als Antwort auf Antigene unter Umgehung der Keimzentrumsreaktion darstellt. Die Lymphknotenarchitektur wird weitgehend beibehalten, jedoch wird die parakortikale Zone durch polymorphe Infiltrate mit kleineren Blasten, Zentroblasten, Immunoblasten und insbesondere Plasmablasten erweitert. Je nach Blastenmorphologie variieren ihre immunphänotypischen Merkmale und zeigen eine heterogene Positivität für CD20, CD30, CD38, CD79a, CD138, IRF4 (MUM1) und BLIMP1 [5]. Diese Blasten sind polytypisch für Leichtketten und sind zu 70–80 % IgM^+^/CD27^-^/CD30^-^/CD79a^+^/CD138^−^, da sie auf der genetischen Ebene keinen Immunglobulinklassenwechsel und auf der physiologischen Ebene keine Keimzentrumsreaktion erfahren. Zu 20–30 % sind diese Blasten IgG^+^/CD27^+^/CD30^±^/CD79a^−^/CD138^+^ und könnten spezifischen, extrafollikulär aktivierten B‑Zellen der Marginalzonen ohne lang anhaltende B‑Zell-Immunantwort entsprechen [9]. Folglich führt dies zu einer Produktion von niederaffinen IgM-Antikörpern ohne Erzeugung eines immunologischen Gedächtnisses. Betroffen sind vor allem schleimhautnahe Lymphknoten und die Milz.

In diesem Beitrag fassen wir Literaturdaten und eigene Beobachtungen zu den Reaktionsmustern der lokoregionären Lymphknoten im Abflussgebiet von COVID-19-Lungen zusammen. Wir diskutieren diese Beobachtungen im Kontext bekannter Genexpressionsprofile und immunologischer Phänomene bei schweren Krankheitsverläufen sowie hinsichtlich ihrer potenziellen Bedeutung für die Entwicklung einer wirksamen Immunantwort und eines immunologischen Gedächtnisses.

## Lymphknoten(histo)pathologie von COVID-19

**Figure 1:**
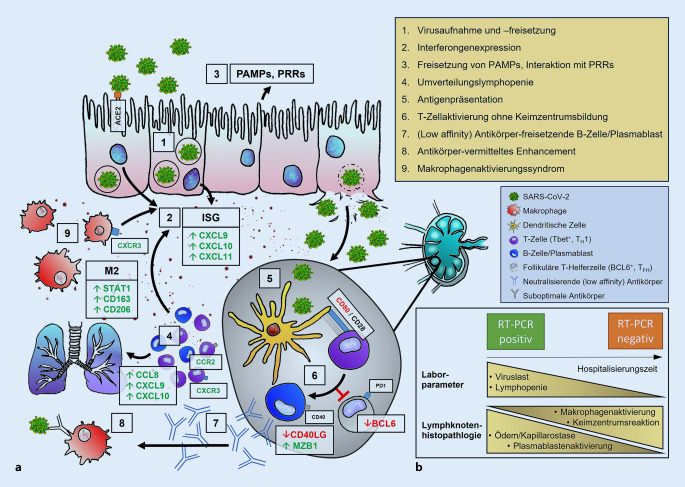

Eine Übersicht der wichtigsten bisher bekannten pathophysiologischen Mechanismen der COVID-19-Immunantwort finden Sie in Abb. 1a.

### Lymphopenie

In ersten klinischen Fallberichten konnte bereits eine schwere Lymphopenie unter COVID-19-Patienten beobachtet werden [16], deren Ausmaß in einer Metaanalyse von 32 Studien mit über 10.000 Patienten der wichtigste prädiktive Faktor des Krankheitsverlaufes darstellte [26]. Die Lymphopenie könnte in Zusammenhang mit Regulationsstörungen von chemotaktisch wirksamen Botenstoffen erklärbar sein. So sind *CCL8, CCL20* und *CXCL10* im Gewebe von COVID-19-Patienten, insbesondere in den Lungen, vermehrt exprimiert [1]. Diese Moleküle sind verantwortlich für die Chemotaxis und Migration von (T-)Lymphozyten, was in Anbetracht der Größe der Lungen unter anderem zu einer Umverteilungslymphopenie führen könnte. An dieser Stelle sei erwähnt, dass Lymphozyten neben anderen Zellen über den Viruseintrittsrezeptor ACE2 verfügen [46], sodass direkte zytopathische Effekte hinsichtlich der Lymphopenie ebenfalls denkbar sind.

### Dysregulation der Interferon-vermittelten Signaltransduktion

Die IFN-vermittelte Signaltransduktion spielt in jeder primären Immunantwort auf virale Infektionen eine zentrale Rolle. Erste Ergebnisse der Einzelzellsequenzierung von mononukleären Zellen aus dem peripheren Blut konnten eine phänotypische Rekonfiguration der Immunzellen mit heterogener IFN-stimulierten Gensignatur (ISG) und Runterregulation der HLA-Klasse-II-Gene bei COVID-19 aufzeigen, was mit einer erhöhten Plasmablastenpopulation assoziiert war [45]. Ferner scheint die ISG-Expression auch mit Krankheitsverlauf und Viruslast zusammenzuhängen [34]. Eine Subgruppe von Patienten mit frühletalem Krankheitsverlauf wies eine hohe Expression von ISG und Zytokinen auf, assoziiert mit hoher Viruslast und diskreter Lungenschädigung (ISG_high_) [30]. Im Gegensatz dazu zeigte eine andere Subgruppe mit vorwiegend protrahiertem Krankheitsverlauf eine verminderte Expression von ISGs (ISG_low_) in Assoziation mit schwerwiegendem diffusem Alveolarwandschaden, niedrigerer Viruslast und einer vermehrten Infiltration von CD8^+^-Zellen und (M2-polarisierten) Makrophagen [34].

### Dysregulation der Antigenpräsentation

Genexpressionsanalysen von COVID-19-Lungen zeigen eine 2fach herrunterregulierte Expression wichtiger kostimulatorischer Moleküle der Antigenpräsentation wie *TLR7, TLR9* und *CD86* [1]. CD86 wird von antigenpräsentierenden Zellen exprimiert und bindet die Liganden CD28 oder CTLA4 an der T‑Zell-Oberfläche. Eine Interaktion zwischen CD86 und CD28 führt zu Stimulation der T‑Zell-Antwort [39]. Weitere TLR (*TLR1, TLR4, TLR5*) sind ebenfalls bei COVID-19 vermindert exprimiert. Während TLR1 an Peptidoglykane grampositiver Bakterien bindet, interagiert TLR4 mit Lipopolysacchariden (LPS) gramnegativer Bakterien und ist zentral für das Erkennen des SARS-CoV-2-Spike-Antigens [36]. Aktivierung von TLR5 durch Flagellin führt zu einer Aktivierung der angeborenen Immunabwehr und ist somit zu Beginn einer Antwort gegenüber flagellierten Bakterien äußerst hilfreich [39]. Dies könnte unter anderem die vermehrte Anfälligkeit für bakterielle Superinfektionen in COVID-19-Patienten erklären.

### Makrophagenaktivierung

Des Weiteren beobachtete man bei COVID-19-Patienten die Entwicklung einer hämophagozytischen Lymphohistiozytose (HLH) [29, 38]. Das unkontrollierte Makrophagenaktivierungssyndrom, welches der HLH zugrunde liegt, gilt als weiterer Ausdruck einer gestörten Antigenpräsentation und exzessiver Zytokinausschüttung („cytokine storm“), ist jedoch – im begrenztem und kontrolliertem Maße – eine typische Reaktion der angeborenen Immunabwehr als „pattern-recognition-receptor“ (PRR)-assoziierte Antwort auf „pathogen associated molecular patterns“ (PAMP), speziell viraler RNA [4, 30, 44]. Infektionsassoziierte HLH können durch Herpesviren, insbesondere EBV, aber auch SARS-CoV‑1, Influenzaviren H5N1 und H1N1 ausgelöst werden [6, 15]. Passend dazu zeigen COVID-19-Gewebeuntersuchungen eine vermehrte Expression von *CCL2* und *CCL7* [1], welche chemotaktisch für Makrophagen wirken, was den Zytokinsturm bei schweren Krankheitsverläufen pathogenetisch zentral aber auch therapeutisch beeinflussbar macht [25]. Tatsächlich beobachtet man bei schweren Krankheitsverläufen die Hochregulierung von Genexpressionsprogrammen der Komplementaktivierung und Phagozytose [48]. Eindrücklich in diesem Zusammenhang ist, dass pädiatrische COVID-19-Patienten eine multisystemische Hyperinflammation entwickeln können, welche phänotypisch dem Kawasaki-Syndrom ähnelt [37]. In solchen Fällen war eine schwere vaskuläre und kardiale Manifestation zu beobachten, vereinzelt zusammen mit einem Makrophagenaktivierungssyndrom.

### Dysregulation der humoralen Immunantwort

In einer der ersten Autopsiestudien wurde histologisch in hilären Lymphknoten und Milzparenchym ein Fehlen von Keimzentren, eine vermehrte Präsenz von Plasmablasten und eine intranodale Kapillarostase beschrieben [30], erklärbar durch eine Dysregulation der BCL6^+^-follikulären T‑Helferzellen [21], welche für die Funktionalität der Keimzentren essenziell sind. Ferner konnte in diesem Zusammenhang eine frühe Blockade der T‑Helferzell-Differenzierung, eine Prädominanz von T‑bet^+^-T-Helfer-1-Zellen und eine extrafollikuläre Akkumulation von TNF‑α gezeigt werden [21], die mit einem Verluste von follikulären B‑Zellen mit durchflusszytometrischen Analysen des peripheren Bluts von schwerkranken COVID-19-Patienten korreliert werden konnte [21]. Interessanterweise sind TLR4 und TLR5, welche beide in COVID-19 herrunterreguliert sind, auch für die Keimzentrumsreaktion essenziell, da sie über MYD88 den NF-κB-Signalweg aktivieren [13].

Die Vermehrung von Plasmablasten in hilären Lymphknoten bei letaler COVID-19-Erkrankung könnte ein morphologisches Korrelat einer Störung des Immunglobulin-(Ig)-Klassenwechsels darstellen. In der Tat zeigen insbesondere Patienten mit schwerem Krankheitsverlauf deutlich vermehrt Plasmablasten, während eine robuste adaptive Immunantwort mit klonal expandierten CD8^+^-Effektor- allenfalls Gedächtniszellen bei milden Verläufen zu beobachten ist [23, 49]. In Genexpressionsanalysen von COVID-19-Autopsiematerial konnte zudem eine Herrunterregulation von *CD40LG* gezeigt werden [1], welches in der physiologischen Immunantwort ein essenzielles Bindeglied der Kommunikation zwischen T‑ und B‑Zellen darstellt und maßgebend die B‑Zell-Reifung beeinflusst [39]. Ein Defekt in CD40LG resultiert in einem Fehlen des Ig-Klassenwechsels, was die präferenzielle extrafollikuläre Proliferation von B‑Zellen begünstigen könnte. Als Abbild der erwähnten 2 Besonderheiten konnte in detaillierten Analysen eine negative Korrelation zwischen der Menge von B‑Gedächtniszellen und der COVID-19-Symptomdauer gezeigt werden [33]. Die Menge dieser Zellen korrelierte mit IgG1 und IgM gegen das SARS-CoV-2-Spikeprotein. Dies spiegelt sich auch in Antikörpertitermessungen [32] und durchflusszytometrischen Analysen wider, welche in Fällen mit schwerem Krankheitsverlauf eine oligoklonale Plasmablastenexpansion (>30 % der zirkulierenden B‑Zellen) [22] gezeigt haben. Dies trug, zusammen mit anderen immunologischen Signaturen, die mit dem Krankheitsverlauf korrelierten, auch zur biostatistischen Einteilung in 3 COVID-19-Immunotypen mit verschiedenen Risikoprofilen bei [28]. Als Bindeglied zur Immunpathologie kann eine unvollendete humorale Immunantwort mit niederaffinen, nicht (ausreichend) neutralisierenden bzw. niedertitrierten Antikörpern zu einem sog. antikörpervermittelten Enhancement führen. Hierbei ermöglichen suboptimale Antikörper die Penetration des Virus in Fc/Komplement-Rezeptor-tragenden Monozyten, Makrophagen und Granulozyten [19]. Tatsächlich weist die Datenlage auf eine Interaktion von Anti-Spikeprotein-Antikörpern und Makrophagen hin, die zumindest bei SARS-CoV‑1 maßgebend zur Lungenschädigung beiträgt [24].

Letztlich konnte auch gezeigt werden, dass eine Spikeproteinreaktivität in über einem Drittel von SARS-CoV-2-naiven Patienten vorhanden ist. Dies impliziert die Präsenz von kreuzreaktiven T‑Zellen, welche mutmaßlich durch Exposition gegenüber anderen Coronaviren entstanden ist und möglicherweise die robustere Immunantwort in manchen Patientengruppen erklären könnte [4].

## Methoden

### Gewebeasservierung und Histologie

Lymphknoten der hilären, mediastinalen und zervikalen Stationen wurden während der Autopsie (*n* = 20) asserviert. Histochemische und immunhistochemische Untersuchungen (HE, IgG, IgM, CD3, CD11c, CD20, CD79a, CD68, CD163, CD206, HLA-DR, SARS-CoV-2-Nukleokapsid-Antigen [polyklonaler Kaninchenantikörper 200-401-A50 von Rockland Immunochemicals, Inc., Gilbertsville, USA, Verdünnung 1:2000]) wurden im Einklang mit akkreditieren SOP-Protokollen des Institutes durchgeführt. Histologische Charakteristika (Anzahl Plasmablasten, Plasmazellen, Ödem/Kapillarostase, Präsenz von HLH und Keimzentren) wurden mittels Ordinalskalen (0–3) ausgewertet (Tab. 1).

**Table 1:** 

		Lymphknoten-RT-PCR		Gesamt (*n* = 20)
		Positiv (*n* = 14)	Negativ (*n* = 6)	
*Klinik/Laborparameter (letzter Wert vor Exitus)*				
Hospitalisierungszeit (Tage; Median; IQR)		6 (3–9)	11 (4–30)	8 (4–12)
CRP (mg/dl; Median; IQR)		176 (100–271)	315 (232–337)	218 (144–280)
Leukozyten, absolut (10^9^/l; Median; IQR)		8,1 (6,3–11,4)	16,0 (8,8–16,0)	8,8 (7,4–13,8)
Lymphozyten, absolut (10^9^/l; Median; IQR)		0,7 (0,4–1,1)	0,5 (0,4–0,96)	0,7 (0,5–1,0)
Neutrophile Granulozyten, absolut (10^9^/l; Median; IQR)		6,7 (3,7–10,1)	7,3 (7,26–10,6)	6,8 (6,3–10,1)
*Histopathologie und Post-Mortem RT-PCR*				
Viruslast, Lunge (SARS-CoV‑2 Genome/10^6^ RNaseP Kopien; Median; IQR)		2149 (57–60581)	123 (0–365)	146 (0–32218)
Viruslast, Lymphknoten (SARS-CoV‑2 Genome/10^6^ RNaseP-Kopien; Median; IQR)		117 (21–10392)	0 (0)	21 (0–3100)
Keimzentren (*n*, %)	Fehlend	9 (64)	3 (50)	12 (60)
	Wenige	4 (29)	2 (33)	6 (33)
	Viele	1 (7)	1 (17)	2 (10)
Plasmablasten (*n*, %)	Fehlend/wenige	9 (64)	3 (50)	12 (60)
	Mäßig	3 (21)	3 (50)	6 (33)
	Viele	2 (14)	0 (0)	2 (10)
Plasmazellen (*n*, %)	Fehlend/wenige	12 (86)	4 (66)	16 (80)
	Mäßig	2 (14)	2 (33)	4 (20)
	Viele	0 (0)	0 (0)	0 (0)
Ödem und Kapillarostase (*n*, %)	Leichtgradig	4 (29)	0 (0)	4 (20)
	Mäßiggradig	7 (50)	6 (100)	13 (65)
	Schwergradig	3 (21)	0 (0)	3 (15)
Hämophagozytoseaktivität (*n*, %)	Fehlend	5 (36)	3 (50)	8 (40)
	Leichtgradig	6 (43)	3 (50)	9 (45)
	Schwergradig	3 (21)	0 (0)	3 (15)

*CRP* C-reaktives Protein, *IQR* Interquartilsabstand

### RT-PCR

Alle Lymphknoten erfuhren eine RT-PCR-Untersuchung zur Bestimmung der Viruslast. Deren Protokoll wurde bereits in unserer vorherigen Autopsiestudie beschrieben [30].

### Genexpressionsanalysen

RNA wurde durch die HTG Molecular Diagnostics, Inc. (Tucson, USA) nach etablierten Protokollen aus 10 µm dicken unbehandelten Paraffinschnitten extrahiert und exakt wie beschrieben unter Verwendung des HTG EdgeSeq Autoimmune-Assays prozessiert und analysiert [31].

### Statistik

Statistische Analysen erfolgten mittels SPSS, Version 25 (IBM, Armonk, USA). Alle Korrelationsanalysen erfolgten mittels Spearman Rho (ρ).

## Resultate

Tab. 1 stellt eine Übersicht von klinischen und histologischen Parametern der 20 untersuchten Fälle dar.

### Klinische Parameter und RT-PCR

In der RT-PCR-Analyse konnte in 14 von 20 untersuchten Lymphknoten SARS-CoV-2-RNA nachgewiesen werden, während 6 Fälle keine detektierbare Viruslast aufwiesen. Interessanterweise war in diesen letzteren Fällen die Hospitalisierungszeit länger (6 vs. 11 Tage), was ähnlich wie bei vorherig durchgeführten Analysen der gleichen Autopsiekohorte [34] auf einen biphasischen Krankheitsverlauf mit progressiver viraler Elimination hindeutet (Abb. 1b). Passend auch dazu die negative Korrelation der pulmonalen Viruslast mit der Hospitalisierungszeit (ρ = −0,776; *p* < 0,0001), die positive Korrelation zwischen pulmonaler und Lymphknoten-Viruslast (ρ = 0,514; *p* = 0,035), die positive Korrelation zwischen Hospitalisierungszeit und absoluter Lymphozytenzahl (ρ = 0,582; *p* = 0,014; nicht direkt aus Tab. 1 ersichtlich) bzw. die negative mit der absoluten Leukozytenzahl (ρ = −0,577; *p* = 0,015).

### Histopathologie

**Figure 2:**
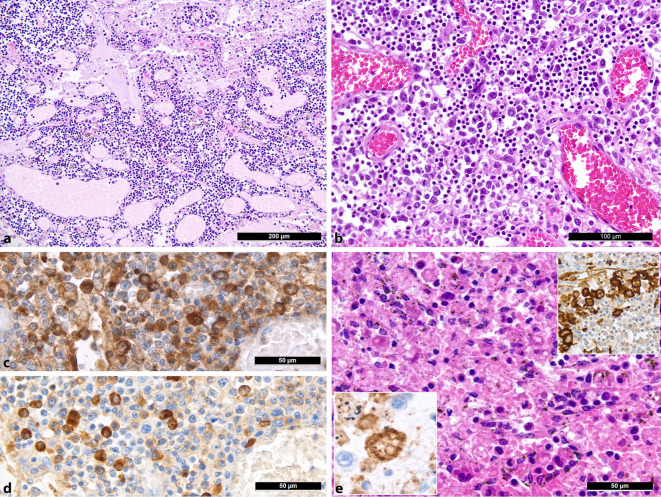

Zu den wichtigsten histologischen Merkmalen zählen ein mäßig- bis schwergradiges Ödem und Kapillarostase (Abb. 2a) in allen Fällen, was primär mit der akuten Rechtsherzbelastung im Rahmen der schweren pulmonalen Erkrankungsbeteiligung erklärbar ist. Besonders auffällig war die Vermehrung von extrafollikulären B‑Blasten, insbesondere IgG- und IgM-positiver Plasmablasten (Abb. 2b, c) in 12 von 20 Fällen, passend zum oben beschrieben Muster einer schnellen bzw. primären und transienten B‑Zell-Immunantwort unter Umgehung der Keimzentrumsreaktion [5] bei weitgehend fehlenden oder hypoplastischen/hypotrophen Keimzentren bzw. Sekundärfollikeln (12 von 20) einschließlich follikulärer dendritischer Zellen und follikulärer T‑Helferzellen. Korrelationsanalysen zeigten eine negative Assoziation zwischen dem Vorhandensein von Sekundärfollikeln und viraler Last der Lunge (ρ = −0,645; *p* = 0,005) und C-reaktivem Protein (Sekundärfollikeln = −0,522; *p* = 0,032), was im Zusammenschau mit vorherigen Analysen der gleichen Autopsiekohorte [34] als Ausdruck einer verspäteten Keimzentrumsreaktion zu interpretieren ist. Ebenfalls fand sich eine diskrete bis mäßiggradige Plasmazytose und eine Prädominanz von CD8^+^-T-Zellen. Augenfällig war die Präsenz von zahlreichen HLA-DR-, CD163- und CD206-positiven M2-polarisierten Makrophagen [24], CD11c- und CD68-positiven Histiozyten und eine wahrnehmbare Hämophagozytoseaktivität in den Sinus in 12 von 20 Fällen (Abb. 2d), im Einklang mit den bekannten Phänomenen der Makrophagenaktivierung bei COVID-19 [29, 38]. Dennoch bot sich das volle klinische Bild einer HLH nur bei einem der 20 hier untersuchten Patienten [47].

**Figure 3:**
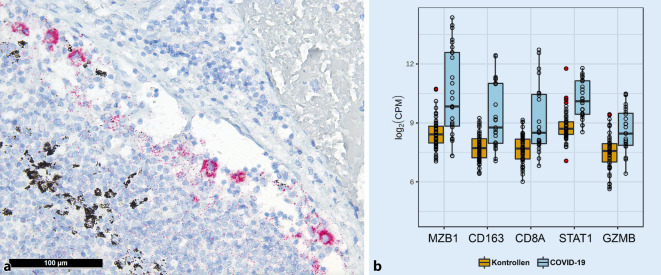

Als eventuellen Ausdruck des oben beschriebenen antikörpervermittelten Enhancements und passend zu den Beobachtungen von Martines et al. [27] konnten wir mittels immunhistochemischen Untersuchungen für das Nukleokapsid-Antigen von SARS-CoV‑2 vermehrte, sich entsprechend positiv färbende Sinushistiozyten bei einer älteren Patientin mit einem 30-tägigen Krankheitsverlauf nachweisen (Abb. 3a). Im Großteil der untersuchten Lymphknoten waren allerdings immunhistochemisch keine signifikanten Antigenmengen feststellbar. Allerdings zeigten die Lungen der gleichen Patientin eine wesentlich höhere Viruskopiezahl (990 Virusgenomkopien/10^6^ humane RNAseP-Kopien in den Lymphknoten und ca. 125.000 in den Lungen).

### Genexpressionsprofile

Im Einklang mit den oben beschriebenen Mustern zeigten Genexpressionsprofile (Abb. 3b) eine vermehrte Expression folgender Gene: *STAT1* (zentraler Transkriptionsfaktor in der Makrophagenaktivierung), *CD163* (Hämoglobin-Haptoglobin-Komplexrezeptor und Marker der M2-Makrophagenpolarisierung [24]), *Granzym B* (aber nicht *Perforin;* passend zur Dysbalance beider Proteine bei der hämophagozytierenden Lymphohistiozytose [40]), *CXCL9* und *PAK1* (ein Chemokin und ein Enzym, welche wichtig für die Migration der zytotoxischen T‑Zellen sind) und – passend dazu – *CD8*, schliesslich *MZB1* („marginal zone B and B1 cell specific protein“), welches – korrelierend mit den vermehrten Plasmablasten – die Zusammensetzung und Sekretion von IgM begünstigt.

## Synthese

Alles in allem scheinen die hier beschriebenen Reaktionsmuster der Lymphknoten bei COVID-19-Patienten mit letalem Krankheitsverlauf die Immunpathologie der Erkrankung abzubilden. Unsere Daten implizieren einen biphasischen Krankheitsverlauf beginnend mit hoher Viruslast und schwergradiger Lymphopenie, welche sich im Laufe der immunologischen Antwort zurückbilden. Korrespondierend sind histologisch in den Lymphknoten von frühletalen Fällen Ödem und Plasmablastenaktivierung, gefolgt in spätletalen Fällen von Makrophagenaktivierung und subtiler Keimzentrumsreaktion zu erkennen (Abb. 1b). Dies steht im Einklang mit Genexpressionsanalysen, welche an der gleichen Autopsiekohorte durchgeführt wurden [34], die eine ausgeprägte Varianz der *ISG*-Gensignatur im Verlauf von COVID-19 gezeigt hat. All dies deutet auf eine Störung der IFN-Antwort hin, welche fundamental für die Abwehr gegenüber intrazelluläre Mikroorganismen, insbesondere Viren, ist:

1. Alle Coronaviren, besonders SARS-CoV‑2, unterdrücken die Produktion und die Freisetzung von allen 3 IFN-tTypen [10].
2. Patienten mit schweren COVID-19-Krankheitsverläufen zeigen Funktionsverlustvarianten in TLR- und IFN-abhängigen Genen oder neutralisierende Antikörper gegen den Typ-I-IFN (α und ω) [3, 49].
3. Die Dysregulation der Typ-I-IFN scheint generell entscheidend für den Krankheitsverlauf zu sein [7, 8].
4. Die ISG-Antwort ändert sich stark im Rahmen des COVID-19-Verlaufs und scheint unterschiedliche Aspekte der Immunpathologie zu beeinflussen [34].

Diese Störung der IFN-Antwort könnte gut die beschriebenen augenfälligen morphologischen Reaktionsmuster der lokoregionären Lymphknoten im Abflussgebiet von COVID-19-Lungen erklären, nämlich die Dysregulation der BCL6^+^-follikulären T‑Helferzellen [21], die rasche, aber wenig spezifische B‑Zell-Immunantwort unter Umgehung der Keimzentrumsreaktion mit Plasmablasten, welche erfahrungsgemäß niederaffine Antikörper herstellen [5, 22, 45], des damit verbundenen antikörpervermittelten Enhancements mit Makrophagen(hyper)aktivierung [19] mit Sinushistiozytose und gegebenenfalls HLH [29, 38] sowie die M2-Makrophagenpolarisierung [24, 34, 49]. Schließlich resultiert diese Dysregulation in eine hochpathogene inflammatorische Monozyten-Makrophagen-Antwort, die bei SARS-CoV‑1 und MERS sehr gut dokumentiert ist [7, 8, 24], welche für einen Großteil der Organschädigung in COVID-19 verantwortlich zu sein scheint [25, 48]. Das antikörpervermittelte Enhancement könnte auch zur *in situ* beobachteten (Abb. 3a) Virusaufnahme in Monozyten/Makrophagen [19] und somit auch zur Virusausbreitung im Körper beitragen.

Die komplexen Wechselwirkungen zwischen SARS-CoV‑2 und dem Immunsystem, welche grob die beschriebenen Reaktionsmuster der Lymphknoten bedingen, könnten zumindest teilweise auch die bekannten Herausforderungen und Probleme bei der Entwicklung von effizienten Coronavirusimpfungen erklären [11]. Andererseits könnten Erkenntnisse aus *In-situ*-Studien des lymphatischen Kompartiments bei COVID-19 wertvolle Ansätze, beispielsweise für den gezielten Gebrauch smarter kleinmolekularer Adjuvantien wie TLR-Agonisten [35], zur Effizienzsteigerung der in Entwicklung befindlichen Vakzinen hinsichtlich Aufbau eines dauerhaften immunologischen Gedächtnisses liefern.

## Fazit für die Praxis

- Das Erforschen histomorphologischer Merkmale in lokoregionären Lymphknoten ist unabdingbar zum Verständnis der Pathophysiologie von COVID-19. Besonders das Ausbleiben der Keimzentrumsreaktion mit Plasmablastenexpansion und fehlendem Immunglobulingenklassenwechsel deuten auf ineffiziente Antikörperreaktion bei Patienten mit schwerem Krankheitsverlauf hin.
- Die Dysregulation der Interferon(IFN)-Antwort scheint zentral für die Immunpathologie von COVID-19 zu sein. Erste Therapieansätze IFN-I-interferierender Medikamente (wie der JAK-Inhibitor Baricitinib oder Dexamethason) werden bereits in klinischen Studien erforscht (NCT04358614), während Studien mit TLR-Agonisten erst in Planung sind.
- Weitere Studien werden benötigt, um die Mechanismen der angeborenen und humoralen Immunantwort zwischen milden und schweren Krankheitsverläufen sowie Phänomene wie die Kreuzreaktivität bestimmter T‑Zellgruppen besser zu verstehen. Dies wird der Entwicklung einer erfolgreichen Impfstrategie möglicherweise behilflich sein.

## Abkürzungen

BLIMP-1/PRDM‑1: „PR domain zinc finger protein 1“
CCL: „Chemokine ligand“
CD: „Cluster of differentiation“
COVID-19: „Coronavirus disease 2019“
CTLA4: „Cytotoxic T‑lymphocyte-associated protein 4“
CXCL9: „Chemokine (C-X‑C motif) ligand 9“
EBV: Epstein-Barr-Virus
Fc: „Fragment constant”
HLA-DR: „Human leukocyte antigen DR“
HLH: „Hemophagocytic lymphohistiocytosis“
IFN: Interferon
Ig: Immunoglobulin
IL: Interleukin
IRF4 (MUM-1): „Interferon regulatory factor 4“
ISG: „Interferon-stimulated genes“
LPS: Lipopolysaccharid
MUM‑1 (IRF4): „Multiple myeloma 1 protein“
MYD88: „Myeloid differentiation primary response 88“
MZB1: „Marginal zone B and B1 cell specific protein“
NF-κB: „Nuclear factor kappa B“
PAK1: „P21 (RAC1) activated kinase 1“
PAMPs: „Pathogen-associated molecular patterns“
PRRs: „Pattern recognition receptor“
qRT-PCR: „Quantitative reverse transcriptase polymerase chain reaction“
SARS-CoV-1/2: „Severe acute respiratory syndrome coronavirus 1/2“
STAT1: „Signal transducer and activator of transcription 1“
TLR: „Toll-like receptor“
TNF‑α: „Tumor necrosis factor alpha“
